# Protein secretion systems in bacterial-host associations, and their description in the Gene Ontology

**DOI:** 10.1186/1471-2180-9-S1-S2

**Published:** 2009-02-19

**Authors:** Tsai-Tien Tseng, Brett M Tyler, João C Setubal

**Affiliations:** 1 Virginia Bioinformatics Institute, Virginia Polytechnic Institute and State University, Blacksburg, VA 24061, USA; 2 School of Biology, Georgia Institute of Technology, Atlanta, GA 30332, USA; 3 Department of Plant Pathology, Physiology and Weed Science, Virginia Polytechnic Institute and State University, Blacksburg, VA 24061, USA; 4 Department of Computer Science, Virginia Polytechnic Institute and State University, Blacksburg, VA 24061, USA

## Abstract

Protein secretion plays a central role in modulating the interactions of bacteria with their environments. This is particularly the case when symbiotic bacteria (whether pathogenic, commensal or mutualistic) are interacting with larger host organisms. In the case of Gram-negative bacteria, secretion requires translocation across the outer as well as the inner membrane, and a diversity of molecular machines have been elaborated for this purpose. A number of secreted proteins are destined to enter the host cell (effectors and toxins), and thus several secretion systems include apparatus to translocate proteins across the plasma membrane of the host also. The Plant-Associated Microbe Gene Ontology (PAMGO) Consortium has been developing standardized terms for describing biological processes and cellular components that play important roles in the interactions of microbes with plant and animal hosts, including the processes of bacterial secretion. Here we survey bacterial secretion systems known to modulate interactions with host organisms and describe Gene Ontology terms useful for describing the components and functions of these systems, and for capturing the similarities among the diverse systems.

## Introduction

Bacteria form a very wide diversity of biotic associations, ranging from biofilms to mutualistic or pathogenic associations with larger host organisms. Protein secretion plays a central role in modulating all of these interactions. With the rapid accumulation of bacterial genome sequences, our knowledge of the complexity of bacterial protein secretion systems has expanded. In Gram-negative bacteria, where secretion involves translocation across inner and outer membranes, there are now known six general classes of protein secretion systems, each of which shows considerable diversity. Gram-positive bacteria share some of the same secretion systems as Gram-negative bacteria and also display one system specific to that group, the type VII system. Many proteins secreted by pathogens and other symbionts have the ability to enter inside host cells to modify host physiology to promote colonization (toxins and effector proteins). Several specialized secretion systems have evolved in Gram-negative bacteria to facilitate this process, while intracellular bacteria that lack an outer membrane such as cell-wall-less mollicutes and the Gram-positive bacteria *Listeria monocytogenes *and *Rhodococcus equi *can achieve this simply via general secretion pathways.

The Plant Associated Microbe Gene Ontology (PAMGO) project has been developing standardized terms for describing biological processes and cellular components that play important roles in the interactions of microbes with each other and with host organisms, including animals as well as plants [1]. The central purpose of these terms is to enable commonalities in function to be identified across broad taxonomic classes of organisms, including both microbes and hosts. An important concept underlying these terms is that they are agnostic of the outcome of an interaction, which can be very context dependent. The term "symbiosis" is used as a general description of any intimate biotic interaction between an organism such as a microbe with a larger host organism. The incorrect usage of symbiosis as a synonym for mutualism is strongly discouraged. Thus most of the PAMGO terms have as their parent "GO:0044403: symbiosis, encompassing mutualism through parasitism". The term "GO:0009405 pathogenesis" can be used when there is unequivocal evidence that a process is deleterious to the host, but no detailed mechanistic terms are listed under "GO:0009405 pathogenesis". This review provides a brief survey of eight classes of secretion systems, then describes Gene Ontology terms that are now available for annotating the secretion machineries, as well as missing terms that still need to be added. The review concentrates on the machinery of the protein secretion systems, rather than on the secreted proteins, which are the subject of two accompanying reviews in this supplement [2,3].

## Secretion systems

**Figure 1:**
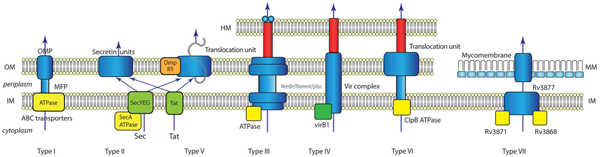
**Summary of known bacterial secretion systems**. In this simplified view only the basics of each secretion system are sketched. HM: Host membrane; OM: outer membrane; IM: inner membrane; MM: mycomembrane; OMP: outer membrane protein; MFP: membrane fusion protein. ATPases and chaperones are shown in yellow.

Figure 1 summarizes the main features of the known secretion systems. In Gram-negative bacteria, some secreted proteins are exported across the inner and outer membranes in a single step via the type I, type III, Type IV or type VI pathways. Other proteins are first exported into the periplasmic space via the universal Sec or two-arginine (Tat) pathways and then translocated across the outer membrane via the type II, type V or less commonly, the type I or type IV machinery. In Gram-positive bacteria, secreted proteins are commonly translocated across the single membrane by the Sec pathway or the two-arginine (Tat) pathway. However, in Gram-positive bacteria such as mycobacteria that have a hydrophobic, nearly impermeable cell wall, called the mycomembrane, a specialized type VII secretion system translocates proteins across both the membrane and the cell wall via a (still poorly-defined) channel, but it is not known yet if this is a one-step or two-step process.

### General secretion and two-arginine (Tat) pathways

The general secretion (Sec) pathway and the two-arginine or Tat translocation pathway are both universal to eubacteria, archaea and eukaryotes (reviewed in [4-6]). In archaea and Gram-positive bacteria the two pathways are responsible for secretion of proteins across the single plasma membrane, while in Gram-negative bacteria they are responsible for export of proteins into the periplasm. The machinery of the Sec pathway recognizes a hydrophobic N-terminal leader sequence on proteins destined for secretion, and translocates proteins in an unfolded state, using ATP hydrolysis and a proton gradient for energy [4]. The machinery of the Tat secretion pathway recognizes a motif rich in basic amino acid residues (S-R-R-x-F-L-K) in the N-terminal region of large co-factor containing proteins and translocates the proteins in a folded state using only a proton gradient as an energy source [5].

A very detailed understanding of the Sec machinery has been developed through 30 years' of genetic, biochemical and biophysical studies, principally in *E. coli *[4]. The protein-conducting pore of the Sec translocase consists of a membrane-embedded heterotrimer, SecY/SecE/SecG (sec61α, sec61β and sec61γ in eukaryotes). The cytoplasmic SecA subunit hydrolyzes ATP to drive translocation. Proteins may be targeted to the translocase via two routes. Membrane proteins and proteins with very hydrophobic signal sequences are translocated co-translationally; the signal sequence is bound by the signal recognition particle, which then targets the ribosome to the translocase via the FtsY receptor. Other secreted proteins are recognized by the SecB chaperone after translation has (mostly) been completed; SecB targets the protein to the translocase by binding to SecA [4].

In *Escherichia coli*, the Tat translocon consists of three different membrane proteins, TatA, TatB, and TatC. TatC functions in the recognition of targeted proteins, while TatA is thought to be the major pore-forming subunit [5].

### Type I secretion system

The type I protein secretion system (T1SS) contains three major components: ATP-binding cassette (ABC) transporters, Outer Membrane Factors (OMFs), and Membrane Fusion Proteins (MFP) [7,8]. While ATP hydrolysis provides the energy for T1SS, additional structural components span the whole protein secretion machinery across both inner and outer membranes. Structurally, OMFs provide a transperiplasmic channel penetrating the outer membrane, while connecting to the membrane fusion protein (MFP) [7,8], which can be found in Gram-positive bacteria [9] as well as Gram-negative bacteria. The MFP is therefore responsible for connecting the OMF and ABC in the periplasmic space.

The proteins making up the ABC exporter component of the T1SS can be divided into two major groups: one specific for large proteins from Gram-negative bacteria and another group for exporting small proteins and peptides. The ABC exporters in T1SS contain two cytoplasmic domains for hydrolysis of ATP and two integral transmembrane domains [7]. In general, the phylogeny of ABC transporters reflects their substrate specificity, implying that shuffling rarely occurred among ABC transporters during their history of evolution [10]. On the other hand, OMFs have not been evolving in parallel with their primary permeases. The evolution of MFPs is in good agreement with the phylogeny of primary permeases [10].

The TolC-HlyD-HlyB complex of *E. coli *has been well-studied for over a decade. TolC is an integral membrane protein on the outer membrane while HlyD (MFP) and HlyB (ABC) occupy the periplasmic space and inner membrane, respectively [7,8]. The substrate in this model system from human uropathogenic strains of *E. coli *is a hemolytic toxin called HlyA [11]. It has been suggested that HlyA must be secreted as an unfolded peptide in a GroEL-dependent fashion [7,8]. Although it has been suggested that a TolC trimer forms a transmembrane channel on the outer membrane, the specific stoichiometry of other components of the type I secretion system remains unclear [7,8]. The outer membrane factor protein, TolC, can also associate with many other transporter families, such as major facilitator superfamily (MFS) and resistance-nodulation-division (RND) superfamily.

Recent studies have identified several examples of the role of the T1SS in the interaction of plant-associated microbes with their hosts [7]. In the rice pathogen *Xanthomonas oryzae *pv. *oryzae *expression of the effector AvrXa21 requires a type I secretory complex composed of RaxA, RaxB and RaxC. Phylogenetic analysis suggested that RaxB functions as an ABC transporter [12], equivalent to HlyB from *E. coli*. It was hypothesized that AvrXa21 molecules consist of a small sulfated polypeptide that is secreted via the type I secretion system and which can be sensed by plant hosts [12]. Virulence factors such as metalloproteases, adhesions and glycanases secreted via the T1SS can also be found in the plant pathogens *Agrobacterium tumefaciens, Pseudomonas syringae *pv *tomato, Ralstonia solanacearum, Xanthomonas axonopodis *pv. *citri *and *Xylella fastidiosa *[7,13].

A common mechanism in the rhizobium-legume symbiosis relies on secreted rhizobial proteins with a novel repeat motif to determine host specificity [7,14]. Some of these proteins are exported via the type I secretion system and are also involved in biofilm formation [15]. It is also possible that type I secretion system can secret exo-polysaccharide in addition to protein for the formation of biofilm. The TolC protein from *Sinorhizobium meliloti *was also found to affect symbiosis [16]. Proteins secreted by the T1SS are also found in *Mesorhizobium loti *and *Bradyrhizobium japonicum *[7].

### Type II secretion system

The type II secretion system (T2SS) is also known as the Sec-dependent system as many proteins that pass through the T2SS must first reach the periplasm via the Sec pathway. Although Sec-dependent translocation is universal [17], the T2SS is found only in the Gram-negative proteobacteria phylum [18,19]. It is found in species that span from obligate symbionts (mutualistic, commensal and pathogenic) to free-living species, but is not universal among any particular group. It appears to be a specialized system that promotes functions specific to the interaction of a species with its biotic or abiotic environment [18,19]. A species may have more than one T2SS [18,19]. The T2SS is required for virulence of the human pathogens *Vibrio cholerae*, *Legionella pneumonphila*, and enterotoxigenic *E. coli*, and of the plant pathogens *Ralstonia solanacearum, Pectobacterium atrosepticum *(*Erwinia carotovora *subsp. *atroseptica*) and *Xanthomonas campestris *pv.*campestris *[18,19]. Virulence determinants secreted via the T2SS include the ADP-ribosylating toxins of enterotoxigenic *E. coli *(heat labile toxin), *V. cholerae *(cholera toxin) and *P. aeruginosa *(exotoxin A) and the pectinases and pectate lyases of the plant pathogens *Dickeya dadantii *(*Erwinia chrysanthemi*), *Erwinia amylovora *and *Xanthomonas campestris *pv.*campestris*. On the other hand, proteobacteria lacking a T2SS include pathogens such as *Agrobacterium tumefaciens*, *Coxiella burnetii *and *Shigella flexneri *and the mutualists *Sinorhizobium meliloti *and *Wolbachia pipientis *[18,19].

The components of the T2SS and their functions have been well characterized in *Klebsiella, Pseudomonas *and *Aeromonas *[18,19]. The translocation pore in the outer membrane is composed of 12–15 secretin subunits – pulD in *Klebsiella oxytoca*, xcpQ and hxcQ in *Pseudomonas aeruginosa*, exeD in *Aeromonas hydrophila*, xpsD in *Xanthomonas campestris*, outD in *Dickeya dadantii *(*Erwinia chrysanthemi*) and in *Erwinia amylovora*. The pore is large enough to accommodate folded proteins such as *P. aeruginosa *elastase (6 nm diameter) [18,19]. The remaining 11–14 conserved components of the T2SS appear to be involved in anchoring of the pore to the inner membrane, and include integral inner membrane subunits, pseudopilin subunits that span the periplasm, and an intracellular ATPase that may provide energy required to regulate the opening and closing of the secretin pore [18,19]. Although the T2SS has an inner membrane component, this component is not involved in translocation of targeted proteins across the inner membrane; this is carried out instead by the Sec and Tat pathways. The structure of the inner membrane complex and the pseudopilins closely resembles that of the type IV piliation system (see type IV secretion, below), suggesting a common evolutionary origin [18,19].

### Type III secretion system

The type III secretion system (T3SS) is found in Gram-negative bacteria that interact with both plant and animal hosts, either as pathogens or mutualists [20-22]. The machinery of the T3SS, termed the injectisome, appears to have a common evolutionary origin with the flagellum [20]. The principal known function of the injectisome is to deliver effector proteins across the bacterial and host membranes into the cytosol of host cells, where they may modulate a large variety of host cell functions, including immune and defense responses (reviewed in [21,22] and in this supplement [2,3]). In some cases however, effector proteins are simply secreted out of the cell. Although initially discovered in pathogenic bacteria, T3SS systems have been identified in rhizobial nitrogen-fixing mutualists of plants, in the tse-tse fly mutualist, *Sodalis glossinidius*, in the nematode mutualist *Photorhabdus luminescens *and in the human commensal *Pantoea agglomerans*, indicating that the T3SS is a hallmark of microbe-host associations, rather than of pathogenesis specifically [20]. Seven families of T3SS machinery have been identified [20]. Plant pathogens are confined to two of these families (Hrp1 and Hrp2) while the T3SS of rhizobial bacteria form a third family. Some bacteria may harbor more than one T3SS; for example *Salmonella typhimurium *contains two pathogenicity islands (SPI-1 and SPI-2), each of which encodes a different T3SS. Although up to 25 proteins may be required to assemble an injectisome, only nine are conserved across all seven families (designated Hrc in the case of plant pathogens), eight of which are also conserved in the flagellar apparatus [20]. Thus there has been considerable divergence and specialization of the T3SS. In many cases, T3SS genes are encoded in pathogenicity islands from foreign sources and/or are located on plasmids, and are commonly subject to horizontal gene transfer [23].

The structure and function of the injectisome have been well studied in the animal pathogens *Salmonella typhimurium *and *Yersinia pestis *and in the plant pathogen *Pseudomonas syringae *(reviewed in [20,24]). The injectisomes are composed of a series of basal rings that span the bacterial inner and outer membranes, connected to a hollow needle (in *Yersinia*), filament (in *Salmonella*) or pilus in (*P. syringae*). Each structure is tipped with a translocation pore that is inserted into the plasma membrane of the target cell [20,24]. A conserved ATPase associates with the bacterial cytoplasmic base of the injectisome and energizes transport. Two classes of chaperones aid in assembly of the injectisome, while a third class assist in translocation of effector proteins [20].

### Type IV secretion system

In comparison to other secretion systems, the type IV secretion system (T4SS) is unique in its ability to transport nucleic acids in addition to proteins into plant and animal cells, as well as into yeast and other bacteria [25]. The T4SS complex can span both membranes of a Gram-negative bacterium or the membrane and the cell envelope of a Gram-positive bacterium. Many organisms have homologous type IV secretion systems, including the pathogens *Agrobacterium tumefaciens *C58 (VirB), *Helicobacter pylori *(CAG; ComB), *Pseudomonas aeruginosa *(TraS/TraB), *Bordetella pertussis *(Ptl), *E. coli *(Tra), *Legionella pneumophila *(Dot) [25] and the nitrogen-fixing plant mutualist *Mesorhizobium loti *[26]. While these systems may share functional similarities, not all systems contain the same sets of genes [27]. The only common protein is VirB10 (TrbI) among all characterized systems [17]. Although type IV secretion systems have garnered attention because of roles in pathogenesis, it is important to point out that not all bacteria have a T4SS.

*Agrobacterium tumefaciens *C58 has been the model system for studying the T4SS. The VirB system from *A. tumefaciens *C58 is capable of exporting DNA-protein complex from its Ti plasmid into the host [25]. The main virulence mechanism is to inject T-DNA into the host to cause cancerous growth or the formation of crown gall tumors, which then produce opines as carbon and energy sources for the pathogen. The major components of the T4SS in *A. tumefaciens *C58 are VirB2-VirB11 and VirD4. VirB1 is responsible for the remodeling of the peptidoglycan via the activity of lytic transglycosylase. The majority of the VirB proteins are responsible for forming the structure complex of the secretory machinery, which is powered by the hydrolysis of ATP.

### Type V secretion system

There are three sub-classes of the type V secretion machinery (T5SS). The archetypal bacterial proteins secreted via the T5SS (and dubbed the T5aSS sub-class) consist of an N-terminal passenger domain from 40 Kd to 400 Kd in size and a conserved C-terminal domain, which forms a beta barrel (reviewed in [28-31]). The proteins are synthesized with an N-terminal signal peptide that directs their export into the periplasm via the Sec machinery. The beta barrel can insert into the outer membrane and is required for translocation of the passenger domain into the extracellular space. In some cases, such as adhesins, the passenger domain remains attached to the beta barrel and the protein remains anchored in the outer membrane. In other cases, the passenger domain is cleaved from the beta barrel and forms a soluble hydrolytic enzyme or toxin. These proteins have been called auto-transporters because the C-terminal domains form a beta barrel with the potential to form a pore through which the N-terminal domain could pass [28-31]. More recent detailed structural studies however suggest that the barrel is incapable of transporting the passenger domain by itself [30]. A helper protein, perhaps Omp85/YaeT, has been hypothesized to facilitate translocation across the outer membrane [30]. A second sub-class of proteins secreted via the T5SS process, dubbed T5cSS proteins, are trimeric proteins in which a single beta barrel is formed by contributions from all three polypeptides. The third sub-class of T5SS proteins, dubbed T5bSS, consists of pairs of proteins in which one partner carries the beta barrel domain, and the other partner is the secreted protein; this process is also called two partner secretion (TPS) [28].

A very large number of proteins are secreted via the T5SS, more even than the T2SS, over 500 in the T5aSS class alone [28-31]. Most of the T5SS secreted proteins characterized to date contribute to the virulence of animal or human pathogens [28-31]. Proteins secreted via the T5SS include adhesins such as AIDA-I and Ag43 of *E. coli*, Hia of *Haemophilus influenzae*, YadA of *Yersinia enteroliticola *and Prn of *Bordetella pertussis*; toxins such as VacA of *Helicobacter pylori*; proteases such as IgA proteases of *Neisseria gonorrheae *and *Neisseria meningitides*, SepA of *Shigella flexneri *and PrtS of *Serratia marcescens*; and S-layer proteins such as rOmpB of *Rickettsia *sp. and Hsr of *Helicobacter pylori*. T5bSS (TPS) secreted proteins include adhesins such as HecA/HecB of the plant pathogen *Dickeya dadantii (Erwinia chrysanthemii) *and cytolysins such as ShlA/ShlB of *Serratia marcescens*, HpmA/HpmB of *Proteus mirabilis *and EthA/EthB of *Edwardsiellla tarda*.

### Type VI secretion system

The type VI secretion machinery (T6SS) is a recently characterized secretion system that appears to constitute a phage-tail-spike-like injectisome that has the potential to introduce effector proteins directly into the cytoplasm of host cells (reviewed in [32-35]), analogous to the T3SS and T4SS machineries. The T6SS machinery was first noticed as a conserved family of pathogenicity islands in Gram-negative bacteria, then was identified as encoding secretory machinery in 2006. More than a quarter of sequenced bacterial genomes contain genes for T6SS components, mostly within the proteobacteria, but also within the planctomycetes and acidobacteria. The T6SS is required for virulence in human and animal pathogens such as *Vibrio cholerae*, *Edwardsiella tarda*, *Pseudomonas aeruginosa*, *Francisella tularensis*, and *Burkholderia mallei*, and also in plant pathogens *such as Agrobacterium tumefaciens, Pectobacterium atrosepticum *and *Xanthomonas oryzae *[32-37]. Furthermore it is required for efficient root colonization by the nitrogen-fixing plant mutualists *Mesorhizobium loti *and *Rhizobium leguminosarum*. Intriguingly, genes encoding the T6SS are also found in some non-symbionts such as *Myxococcus xanthus*, *Dechloromonas aromatica *and *Rhodopirellula baltica*, where it may contribute to environmental adaptation such as biofilm formation. Based on a synthesis of the available experimental evidence, as well as sequence similarities with some components of the T4SS and of the tail spike complex of T4 phage, a model of the T6SS injectisome was proposed that includes a cytoplasmic chaperone with ATPase activity, a channel bridging from the inner to the outer membrane, and a needle tipped with a pore-forming protein [33]. Some components of the machinery may also act as effectors, translocated into host cells. For example VgrG-1, which is a component of the *Vibrio cholerae *T6SS, contains a C-terminal domain that can enter macrophages where it cross-links actin [38]. Overall however, the identities and functions of T6SS effectors are still poorly understood.

### Type VII secretion system

Although Gram-positive bacteria have only a single membrane, some species, most notably the mycobacteria, have a cell wall that is heavily modified by lipids, called a mycomembrane. As a result, the genomes of these species encode a family of specialized secretion systems collectively called type VII section systems (T7SS) (reviewed in [39]). The presence of the T7SS was initially predicted bioinformatically based on clustering of genes encoding secreted proteins that lacked signal sequences with those encoding membrane proteins, ATPases and/or chaperones. Sequencing of the *Mycobacterium bovis *BCG vaccine strain, and mutational analysis of the ESX-1 cluster in *M. tuberculosis *confirmed the hypothesis. ESX-1 is also required for virulence and hemolysis in the fish pathogen *Mycobacterium marinum*, and for conjugation in the non-pathogenic species *Mycobacterium smegmatis *[39]. Mycobacterial genomes contain up to five T7SS gene clusters that do not functionally complement one another. T7SS gene clusters are also found in the closely related pathogens *Corynebacterium diphtheriae *and *Nocardia *[39]. More distantly related gene clusters are also found in the genomes of pathogenic and non-pathogenic Gram-positive species that lack mycomembranes such as *Streptomyces *species and firmicutes such as *Bacillus *and *Clostridium *spp., *Staphylococcus aureus*, *Streptococcus agalactiae *and *Listeria monocytogenes*. The T7SS is required for virulence in *Staphylococcus aureus *but not in *Listeria monocytogenes *[39].

The structure and operation of the T7SS are still being pieced together. Current models [39] suggest an inner membrane translocation channel formed by the integral membrane protein Rv3877, and a separate channel in the mycomembrane composed of as yet unknown protein(s). Chaperone-like ATPases anchored to the inner membrane bind the C-termini of effectors, which are invariably secreted as heterodimers.

## How the Gene Ontology addresses secretion systems

In this section we review the GO terms that were specifically created by the PAMGO project for secretion systems. Many of the functions and processes of proteins related to secretion systems (for example effectors) can be described with GO terms from other parts of the GO hierarchy; those are not covered here in detail. We also note that many additional terms are still needed in this area, especially for secretion systems that are not central to bacteria-host interactions and which therefore have received less attention from the PAMGO consortium.

The following schema illustrates the parallel nature of the GO terms for each of the secretion systems:

### Component ontology

• type (I–IV, VI) protein secretion system complex (Type VII currently missing; type V does not exist as a distinct identifiable complex)

### Process ontology

• children of "GO:0009306 protein secretion":

o protein secretion by the type (I–VI) secretion system (Type VII missing)

• descendants of "GO: 0052047 interaction with other organism via secreted substance during symbiotic interaction"

o "interaction with host via protein secreted by type II/III/IV secretion system"

o "modification of morphology or physiology of other organism via protein secreted by type II/III/IV secretion system during symbiotic interaction"

o both of these have as child:

▪ "modification by symbiont of host morphology via protein secreted by type II/III/IV secretion system"

**Figure 2:**
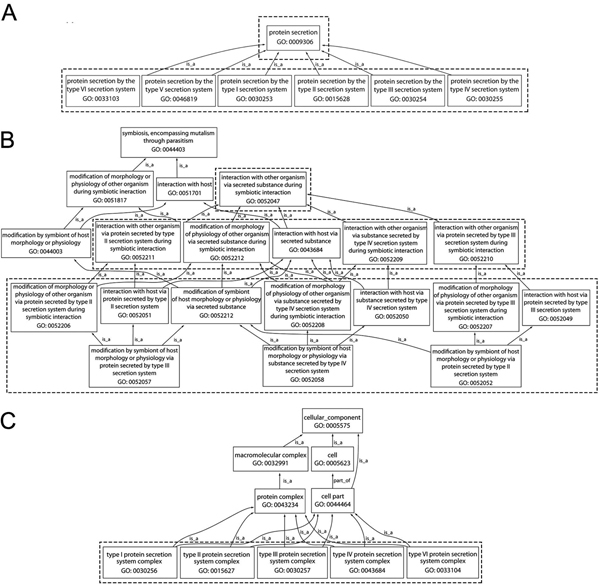
**Gene Ontology terms for secretion systems under "cellular component" and "biological process."**. GO terms for secretion systems described in this review article are encased in dashed boxes. (A) shows terms that are children of the process term "GO ID: 0009306 protein secretion". (B) shows terms that are children of the process term "GO:0044403: symbiosis, encompassing mutualism through parasitism". (C) shows terms that are children of "GO ID: 0005575 cellular component".

The terms above are depicted in Figure 2. Note that in the component ontology and among the children of "GO:0009306 protein secretion" there is just one term for each secretion system; hence the use of such terms is straightforward and perfectly parallel for all secretion systems that have been addressed so far by the PAMGO consortium. Currently, detailed descendant terms of "GO: 0052047 interaction with other organism via secreted substance during symbiotic interaction" are available only for systems II, III, and IV. However, as noted in the survey of secretion systems above, examples exist in which organism interactions are modulated by proteins secreted via systems I, V, VI and VII as well as via the universal Sec and Tat pathways. Thus the PAMGO consortium is currently creating parallel terms for these six systems. Note also that no system-specific terms have yet been created in the molecular function ontology.

The family of terms "Interaction with host via protein secreted by type *number *secretion system" is appropriate for annotating gene products that form the apparatus of secretion when there is experimental evidence that the interaction with the host is affected by secretion through that apparatus. As an example (once terms for the T7SS have been created), in mycobacterial pathogens that contain multiple T7SS gene clusters, if deletion of a cluster affected virulence then the gene in the cluster could be annotated with "Interaction with host via protein secreted by type VII secretion system". However, if deletion of a different cluster did not affect virulence then the term would not be appropriate for that cluster and only the term "protein secretion by the type VII secretion system" would be appropriate. Similarly, in a non-pathogen such as *Mycobacterium smegmatis*, none of the T7SS systems should be annotated with "Interaction with host via protein secreted by type VII secretion system", even if a particular T7SS gene cluster were orthologous to a T7SS in a pathogenic species. On a similar theme, if experimental evidence shows that a gene or gene cluster is important to symbiosis, it may be annotated with "Interaction with host via protein secreted by type *number *secretion system", even if some genes in the cluster appear to be pseudogenes; thus experimental evidence takes precedence over bioinformatic inferences.

The family of terms "modification of morphology or physiology of other organism via protein secreted by type *number *secretion system during symbiotic interaction" and "modification by symbiont of host morphology via protein secreted by type *number *secretion system" are appropriate for annotating the effector proteins that are transported by the secretion systems, but not for the components of the secretion system itself. On the other hand, there are many cases where proteins have a dual function as part of the transport machinery and as effectors. The most striking of these is the "autotransporter" proteins that are secreted via the T5SS pathway in which an N-terminal effector domain is fused to a C-terminal transporter domain. Some proteins associated with the T6SS also appear to be similarly bi-functional [38].

A common theme among most of the secretion systems is the role of ATP hydrolysis and chaperones (Figure 1). This is not yet captured in a systematic way in the GO. Nevertheless the following terms are appropriate in this context: "GO: 0015450 P-P-bond-hydrolysis-driven protein transmembrane transporter activity" and "GO: 0016887 ATPase" and "GO:0042623 ATPase activity, coupled", while "GO: 0043190 ATP-binding cassette (ABC) transporter complex" would be appropriate for the T1SS.

The T2SS and T5SS (and in certain cases T4SS and T1SS as well) deserve a special note because of their relationship with the Sec and Tat pathways. As noted in the first part of this article, proteins translocated via T2SS or T5SS (and sometimes the T1SS and T4SS) first go through the Sec or the Tat pathways. GO provides two pairs of parallel terms for the component and process aspects of the Sec and Tat pathways. "GO:0031522 cell envelope Sec protein transport complex" (component) and "GO:0043934 protein transport by the Sec complex" (process) are available for the Sec pathway; and "GO:0033281 Tat protein transport complex" (component) and "GO:0043935 protein transport by the Tat complex" (process) are the corresponding terms for the Tat pathway. As an example, a protein that affects the interaction with a host that is eventually translocated by the T2SS but goes through the Sec pathways first, could be annotated using both "GO: 0052051 Interaction with host via protein secreted by type II secretion system" and "Interaction with host via protein secreted by Sec secretion system"

## Conclusion

Bacteria use a diversity of machinery to secrete proteins as a means for interacting with their environment, which includes the biotic environment of a host in the case of symbionts. Nevertheless there are several common themes in the action and roles of these secretion systems and the terms in the GO, including those added by the PAMGO consortium, are useful for identifying those common themes. The more that these terms are used and added to by the community, the more useful they will be for comparing secretion systems across diverse bacteria.

## Competing interests

The authors declare that they have no competing interests.

## Authors' contributions

The authors contributed equally to this work.
